# Illness perceptions, depression and anxiety in informal carers of persons with depression: a cross-sectional survey

**DOI:** 10.1007/s11136-018-2009-y

**Published:** 2018-09-22

**Authors:** Josianne Scerri, Therese Saliba, George Saliba, Christian A. Scerri, Liberato Camilleri

**Affiliations:** 1 Department of Mental Health, Faculty of Health Sciences, University of Malta, Msida, Malta; 2 Faculty of Health, Social Care and Education, Kingston University and St George’s University of London, London, UK; 3 Mount Carmel Hospital, Attard, Malta; 4 Department of Mental Health, Faculty of Health Sciences, University of Malta, Msida, Malta; 5 Learning Institute for Health Care Professionals, Mosta, Malta; 6 Faculty of Medicine and Surgery, University of Malta, Msida, Malta; 7 Statistics & Operations Research, Faculty of Science, University of Malta, Msida, Malta; 8 Faculty of Health Sciences, University of Malta, Room 51, Msida, MSD 2080 Malta

**Keywords:** Informal carers, Depression, Illness perceptions, Psychological well-being, Common-sense model, Quantitative

## Abstract

**Purpose:**

To examine the illness perceptions of informal carers of persons with depression, using the theoretical framework of Leventhal’s Common-Sense Model (CSM) and to determine whether these illness perceptions are predictors of anxiety and depression, as measures of psychological well-being.

**Methods:**

A cross-sectional survey was conducted with 94 Maltese individuals caring for a person with depression within a community setting. The informal carers completed the modified Illness Perception Questionnaire (IPQS-Relatives version) and the Hospital Anxiety and Depression Scale (HADS). Data were analysed using descriptive statistics, Spearman’s rank order correlations and ANCOVA regression models, to identify predictors of anxiety and depression respectively in the informal carers.

**Results:**

The informal carers perceived depression as a cyclical condition, having negative consequences on both the patient and on themselves. Participants perceived the causes of depression to be mainly psychosocial in nature and generally viewed the treatment as effective. Caring for a person with depression was perceived as having a considerable negative emotional impact on them. Years of caring was identified as a predictor of anxiety accounting for 20.4% of the variance, and timeline chronicity beliefs, consequences (relative) and illness coherence were identified as predictors of depression, accounting for 56.8% of the variance.

**Conclusion:**

Illness cognitions are significant predictors of depression, thereby suggesting that cognition-based interventions may be effective in targeting depression in these informal carers. Thus, health professionals should explore the carers’ personal understanding of the disease, their timeline beliefs and the perceived consequences of providing care, as they relate to their psychological well-being.

## Introduction

According to the World Health Organisation, depression is considered a global public health concern being a significant contributor to the global burden of disease [1]. By the year 2020, it is estimated that depression will rank in second place for global burden of disease [2], moving into first place by the year 2030 [3]. The rising prevalence of depression, coupled with a trend towards the deinstitutionalisation of persons with a mental illness, places an onus of responsibility on their informal carers [4] who provide personal care, practical assistance and emotional support to the care recipient [5]. In the above scenario, informal carers often experience significant levels of distress and perceive themselves as lacking the necessary knowledge and skills to provide effective care [6]. Moreover, the demands faced by these carers may not only have a detrimental effect on their health, but may also influence their decision to abandon care [7]. Hence, it is crucial to examine the illness perceptions of these informal carers as they have been identified as better predictors of their behavioural and emotional outcomes, than the illness severity of the care recipient [8].

The present study applies ‘Leventhal’s Common-Sense Model of Self-Regulation (CSM) [9] to examine the illness perceptions of informal carers of persons with depression. This model was selected as it recognises the role of the influential person (carer) in the life of the patient as a resource of information and therefore highlights the need to examine their perceptions. Although the CSM has been applied to examine the perceptions of carers for various mental health conditions such as schizophrenia [10–12], anorexia nervosa [13] non-affective psychotic disorders [14], and psychosis [15], to date no research has been conducted on the illness perceptions (as highlighted by the CSM) of informal carers for persons with depression. This model posits that individuals generate perceptions regarding the illness based on concrete and abstract sources of information. These beliefs are formed from three main sources of information: (i) external sources such as family, friends and health providers; (ii) lay information that the individual has previously assimilated and the (iii) current experience of the illness [16]. According to the CSM, individuals carry out parallel processing of both cognitive and emotional representations of the illness, which influence their coping strategies and the appraisal of their effectiveness. Originally, these cognitive representations consisted of the following five dimensions: identity, causes, consequences, control/cure and timeline (acute/chronic) [17]. However, additional dimensions were introduced, namely illness coherence, treatment control, personal control, timeline cyclical and emotional representations.

Hence, the present study addresses the dearth in literature, by examining the illness perceptions of carers of persons diagnosed with depression and by determining whether these illness perceptions are predictors of anxiety and depression. This information is of importance as illness perceptions are highlighted as key targets for interventions [8] due to their influence on the well-being of the informal carer and the care recipient [18].

## Methods

### Study design

A cross-sectional survey was conducted using validated tools which provide measures of illness perceptions, anxiety and depression in the informal carers of persons clinically diagnosed with depression. Additional questions were provided examining socio-demographic characteristics of the participants, such as gender and years of care provision to the person with depression.

### Sample

The sample consisted of 94 Maltese participants who provided informal care to persons clinically diagnosed with depression. The majority of respondents were females (*n* = 67, 71.3%) and the modal category for age was 40–50 years. The majority of carers were the spouse/partner (*n* = 38, 40%), followed by siblings (*n* = 26, 28%), significant others (i.e. children/close friends, *n* = 18, 19%) and parents (*n* = 12, 13%), respectively. More than half of the informal carers were in full-time employment (*n* = 54, 57%), with 20% (*n* = 19) having part-time employment and 23% (*n* = 21) being unemployed.

Inclusion criteria consisted of informal carers who were aged 18 years and over and who were caring for a person clinically diagnosed with depression within a community setting .

### Assessment measures

#### Illness Perception Questionnaire for Schizophrenia-Relatives version (IPQS-Relatives; Lobban et al. [12])

Carers’ perceptions for persons with depression were measured using a modified version of the Illness Perception Questionnaire-revised version (IPQ-R) [19]. The IPQ-R developed by Moss-Morris et al. [19] assesses illness perceptions in relation to the following domains: identity (i.e. symptoms associated to the illness), consequences, treatment and personal control, illness coherence (i.e. understanding of the illness), causes and timeline.

Lobban et al. [12] modified the IPQ-R for use with carers of persons having schizophrenia. This modified tool, (i.e. Illness Perception Questionnaire for Schizophrenia-Relatives version, IPQS-Relatives) [12], has been used in studies exploring the illness perceptions of carers of persons with psychosis and eating disorders [20]. General modifications incorporated in the IPQS-Relatives include replacing the word ‘illness’ with ‘mental health problem’. In addition, for the identity subscale, the respondents had to indicate whether the symptom was related to the mental illness, or a side effect of their medication or due to some other reason. As these modifications appear relevant to carers of persons with depression, this modified tool was consequently used in the present study. However, as recommended by the authors of the IPQ-R, researchers should adapt the tool subscales to the particular illness being investigated [19]. Consequently, respondents in the present study were asked to identify symptoms relating specifically to **depression** on a dichotomous scale (i.e. yes/no). For statements in the remaining subscales, respondents indicated their level of agreement on a 5-point Likert scale with 1 (strongly disagree) to 5 (strongly agree).

The subscales examined in this study were

(i) Identity (30 items)—label and symptoms that the carer associates with depression.
(ii) Cause (20 items)—carer’s perceptions regarding the causes of depression.
(iii) Timeline-acute/chronic (6 items)—carer’s perception regarding the duration of depression. A high score relates to perceptions of a more chronic timeline.
(iv) Timeline-cyclical (4 items)—belief that depression and its symptoms are cyclical in nature. A high score represents the perception of a more cyclical pattern of mental health problems over time.
(v) Consequences-carer (9 items)—the carer’s beliefs about the severity of depression and the likely impact on functioning. A high score represents greater perceived negative consequences for the carer.
(vi) Consequences-patient (11 items)—the carer’s beliefs about the severity of depression and the likely impact on the life of the care recipient. A high score reflects greater perceived negative consequences for the care recipient.
(vii) Treatment control (5 items)—belief that the treatment is an effective means to control depression. A high score indicates that carers perceive the treatment for depression as effective in alleviating mental health problems.
(viii) Personal control—carer (4 items)—carer’s perception about his/her own ability to control symptoms. A high score indicates a greater perception of control by the carer.
(ix) Personal control—patient (4 items)—perception about the care recipient’s ability to control his/her symptoms. A high score indicates a greater perception of control by the patient.
(x) Illness coherence (5 items)—degree to which the carer believes that s/he has a coherent understanding about depression. A high score represents perceiving no coherent understanding of the mental health problems.
(xi) Emotional representations (9 items)—the perceived emotional state that the carer associates with depression. A high score reflects a strong negative emotional response in the carer to providing support to a person with depression.

The IPQS-R was also shown to be a reliable and valid measure of illness perceptions with a sample of carers of persons with schizophrenia [12]. The internal consistency values for the subscales are adequate, with Cronbach’s α values ranging between .63 and .83. Test–retest reliability scores were assessed over a two week (*r*_s_ = .62–.91) and 6 month period (*r*_s_ = .53–.88) and were demonstrated to remain stable over time. Concurrent validity was also confirmed through correlational analysis of the subscales with measures of symptom severity, emotional state and attitudes towards adherence to medication.

#### The Hospital Anxiety and Depression Scale (HADS)

The Hospital Anxiety and Depression Scale (HADS) [21] was used to measure anxiety and depression in the carer. This scale is a 14-item questionnaire consisting of seven items related to depression and seven related to anxiety symptoms. Each question has four possible responses, and these are scored on a scale from 0 to 3. The HADS provides an individual anxiety and depression score where the overall possible score ranges from 0 to 21 in both. Furthermore Zigmond and Snaith [21] suggested that an overall score of 0–7 for anxiety/depression falls within the normal limits. A score of 8–10 indicates mild symptoms, while a score of 11 and above suggests moderate and severe symptoms of anxiety/depression. Furthermore the HADS-Anxiety Questionnaire has been demonstrated to have an optimal cut-off score of 8 (sensitivity 0.89, specificity 0.75), while HADS-Depression was found to have an optimal cut-off score of 8 (sensitivity 0.80, specificity 0.88) [22]. Both concurrent and construct validities for the HADS have been demonstrated in studies on individuals with chronic illnesses, as well as for carers [23].

#### Recruitment

The informal carers were approached by nurses working in community mental health clinics. The nature of the study was explained to them and they were provided with an information letter providing details regarding the aim, objectives, content and estimated time required to complete the questionnaires. Participants were also informed that the questionnaires were to be filled in anonymously and that they could refuse to answer any question and withdraw participation at any stage.

Those carers who indicated their willingness to participate were provided with two questionnaires (i.e. the modified IPQS-Relatives and the HADS) to complete. Those informal carers who were willing to participate were instructed to return the completed questionnaires to the researcher in the self- addressed envelope provided.

#### Ethics

Ethical approval was granted for this research by the relevant university research and ethics committee. Participation was voluntary, and the carers were free to withdraw from the study at any time. Although the eventuality of any risks to participants was minimal, the services of a clinical psychologist was available if requested. In addition, respondents were provided with a list of organisations and community health services which could be contacted should they experience any distress due to participation in this study. As the participants filled in the questionnaires anonymously, this was taken as indicating their informed consent.

### Data analysis

The data were analysed using the facilities of SPSS version 19. Missing data were identified in four cases and these were excluded from the present study. Pearson correlation coefficients (*ρ*) were used to examine intercorrelations between illness perception subscales. Moreover, regression analysis was used to identify significant predictors for the outcomes measures of anxiety and depression. These regression models were fitted on the merit that the distributions of the dependent variables (anxiety and depression) were fairly normal and the predictors consisted both of covariates (variables having a metric scale) and fixed factors (categorical variables). An ordinary least squares (OLS) procedure was used to estimate the regression parameters, which is equivalent to a maximum likelihood (ML) procedure in a homoscedastic linear regression model. The final model was identified using a backward elimination procedure. A 0.01 level of significance level was adopted throughout to reduce the probability of Type 1 errors. Several diagnostic tests were used after fitting the regression models to identify outliers, influential observations and other model misspecifications. Studentized deleted residuals were used to identify outliers, where values larger than 2 or smaller than − 2 indicated observations that did not comply well with the model fit. Cook’s distances were used to identify influential data points, where large values indicated observations that have undue impact on the parameter estimates of the regression model. Leverages were used to identify data points locations of which are considerably distant from the data centroid, where leverages larger than the 2p/n threshold value indicated considerable departure from centroid (p is the number of estimated parameters and n is the sample size). Furthermore, multicollinearity between the continuous predictors was tested using collinearity diagnostics, including the condition index and the variance inflation factor (VIF).

## Results

### Identity

Scores for the identity subscale ranged from 9 to 25 with a mean of 15.82 symptoms (SD = 2.53) perceived as associated with depression. Table 1 presents the ten symptoms most frequently attributed to depression and the number and percentage of participants attributing the symptoms to: (i) the actual illness itself (i.e. depression), (ii) side effects of medication and (iii) other factors.

**Table 1:** Symptom attributes to the illness (depression), medication and other factors

Symptom	Number (*N*, %) of participants associating symptom to depression	Symptom attributes (%)		
		Depression as an illness (*n*/%)	Side effect due to medication (*n*/%)	Other factors (*n*/%)
Lack of energy	92 (97.9)	25 (26.6)	65 (69.1)	4 (4.3)
Gaining weight	92 (97.9)	4 (4.3)	86 (91.4)	4 (4.3)
Loss of motivation	90 (95.7)	82 (87.2)	8 (8.5)	4 (4.3)
Being withdrawn	89 (95.0)	82 (87.2)	7 (7.5)	5 (5.3)
Worrying	89 (94.7)	86 (91.5)	2 (2.1)	6 (6.4)
Difficulty concentrating	89 (94.7)	49 (52.1)	34 (36.2)	11 (11.7)
Loss of selfconfidence	89 (94.7)	86 (91.5)	0 (0.0)	8 (8.5)
Feeling worthless	89 (94.7)	88 (93.6)	0 (0.0)	6 (6.4)
Sleeping a lot	88 (93.6)	8 (8.5)	79 (84.0)	7 (7.5)
Loss of interest in personal care	85 (90.4)	79 (84.0)	2 (2.2)	13 (13.8)

### Causes

Table 2 lists the causal attributes endorsed by participants as relating to depression. Participant responses endorsing (i.e. ‘strongly agree’ and ‘agree’) and non-endorsing (i.e. ‘strongly disagree’ and ‘disagree’) the causal attribute, were collapsed, respectively together, to aid in the interpretation of data. The most commonly attributed causes of depression were ‘stress/worry’ (*n* = 88, 93.6%) and ‘thinking too much’ (*n* = 88, 93.6%) followed by their ‘personality’ (*n* = 84, 89.4%), ‘negative mental attitude’ (*n* = 81, 86%) and their ‘own behaviour’ (*n* = 71, 75.2%), respectively. These causal items mainly relate to the person’s behaviour and/or thought processes. Biological causes such as chemical imbalances in the brain were cited by slightly more than half of the participants (*n* = 52, 54.8%). Overall carers did not highly rank traumatic events such as a ‘shocking experience in life’ (*n* = 23, 24.8%), ‘death of a loved one’ (*n* = 12, 12.9%) and ‘money worries’ (*n* = 13, 14%) as causes of depression.

**Table 2:** Causal attributes for depression

Causes	Agree/strongly agree *n* (%)	Undecided *n* (%)	Disagree/strongly disagree *n* (%)
Stress/worry	88 (93.6)	5 (5.3)	1 (1.1)
Hereditary	58 (61.3)	17 (18.3)	19 (20.4)
Diet/eating habits	1 (1.1)	1 (1.1)	92 (97.8)
Poor medical care	7 (7.6)	11 (11.8)	76 (80.6)
Patient’s own behaviour	71 (75.2)	13 (14.0)	10 (10.8)
My own behaviour	3 (3.2)	1 (1.1)	90 (95.7)
Negative mental attitude	81 (86.0)	4 (4.3)	9 (9.7)
Family problems	25 (26.8)	14(15.1)	55 (58.1)
Overwork	10 (10.7)	7 (7.7)	77 (82.6)
Alcohol	6 (6.5)	7 (7.5)	81 (86.0)
Their personality	84 (89.4)	7 (7.4)	3 (3.2)
Brain damage	5 (5.4)	4 (4.3)	85 (90.3)
Lack of friends	1 (1.1)	8 (8.6)	85 (90.3)
Chemical imbalance in brain	52 (54.8)	22 (23.7)	20 (21.5)
Trauma/shocking experience in life	23 (24.8)	9 (9.7)	62 (65.5)
Death of a loved one	12 (12.9)	12 (12.9)	70 (74.2)
Money worries	13 (14.0)	13 (14.0)	68 (72.0)
Lack of sleep	3 (3.3)	3 (3.3)	88 (93.4)
Thinking too much	88 (93.6)	2 (2.1)	4 (4.3)
Their upbringing	12 (13.1)	12 (13.0)	70 (73.9)

Descriptives for each of the illness perception subscales and the subscale midpoint are presented in Table 3.

**Table 3:** Descriptives for the illness perception subscales by carer response

Illness dimension subscale	Theoretical range and subscale (midpoint)	Mean score (S.D.)	Median	Minimum–maximum score
Timeline chronic/acute	6–30 (18)	17.8 (4.9)	16.0	9–28
Timeline cyclical	4–20 (12)	17.8 (.91)	16	12–19
Treatment control	5–25 (15)	18.1 (4.3)	19	10–25
Illness coherence	5–25 (15)	13.7 (3.3)	13	7–21
Emotional representation	9–45 (27)	32 (3.2)	32	23–42
Consequences patient	11–55 (33)	38.7 (4.3)	39.0	30–50
Personal control patient	4–20 (12)	14.9 (2.1)	16.0	10–18
Consequences carer	9–45 (27)	27.7 (3.7)	28	27.9
Personal control carer	4–20 (12)	13.5 (3.0)	15	5–18

*SD* standard deviation

Participants perceived depression to be a cyclical condition (timeline cyclical median score = 16), having negative consequences both on the carer (consequences carer median score = 28) and even more on the care recipient (consequences patient median score = 39). Depression was perceived as controllable both by treatment (treatment control median score = 19) and personally by the carer (personal control carer median score = 15) and care recipient (personal control patient median score = 16). Participants perceived having an understanding of depression (illness coherence median score = 13). On the other hand, the provision of support to the care receivers was perceived as having a negative emotional impact (emotional median = 32) on informal carers.

Table 4 presents the intercorrelations obtained for the illness dimensions using Spearman’s rank order correlations. The significant bivariate correlations, on average, were identified as moderate to high.

**Table 4:** Intercorrelations between IPQ subscales (*N* = 94)

Illness dimension subscales	1	2	3	4	5	6	7	8	9	10
1. Identity	*1.000*	.247	.*206*	− .349*	.291*	.263*	.419*	.397*	− .*098*	− .358*
2. Timeline chronic	.247	*1.000*	.*006*	− .787^*^	.523^*^	.*209*	.606^*^	.453^*^	− .*116*	− .731^*^
3. Timeline cyclical	.206	.006	1.000	− .024	− .032	.054	.055	.021	.229^*^	.006
4. Treatment control	− .349*	− .787*	− .024	1.000	− .578*	− .282^*^	− .685*	− .638^*^	.121	.793*
5. Illness coherence	.291*	.523^*^	− .032	− .578^*^	1.000	.400*	.571*	.555^*^	− .222*	− .588*
6. Emotional	.263*	.209	.054	− .282*	.400*	1.000	.202	.365*	− .121	− .357^*^
7. Consequences patient	.419*	.606*	.055	− .685^*^	.571*	.202	1.000	.597*	− .128	− .641*
8. Consequences carer	.397*	.453*	.021	− .638*	.555*	.365*	.597*	1.000	− .041	− .593*
9. Personal control patient	− .098	− .116	.229	.121	− .222	− .121	− .128	− .041	1.000	.*221*
10. Personal control carer	− .358*	− .731*	.006	.793*	− .588*	− .357*	− .641*	− .593*	.221*	1.000

1 = Identity; 2 = timeline acute/chronic; 3 = timeline cyclical; 4 = treatment control; 5 = illness coherence; 6 = emotional representations; 7 = consequences (patient); 8 = consequences (carer); 9 = personal control (patient); 10 = personal control (carer). Correlations in italics are significant at *p ≤ 0.01 level (2 tailed)

Results demonstrate that attributing more symptoms to depression (identity dimension) was related to perceptions of greater negative consequences for the carer and patients, less knowledge about depression and perceived control by the relative and perceptions that the treatment is less effective.

Greater perceived consequences for the patient and carer and perceiving the treatment to be less effective were all associated with having a poorer understanding of the illness; attributing more symptoms to depression; being more negatively affected emotionally and with less personal control in the informal carer. A stronger perception of the chronic nature of depression was associated with poorer personal control beliefs (carer) and both were associated with greater negative consequences (carer and patient) and emotional impact; a poorer understanding about depression and perceiving the treatment to less effective. Poorer perceived knowledge about the illness (illness coherence) was associated with perceptions of a more chronic timeline for the disease, greater emotional impact and perceived consequences (patient and relative) and decreased perceptions of control (relative).

A regression model was then fitted to identify significant predictors for psychological well-being, namely anxiety and depression. The predictors consisted partly of covariates (variables having a metric scale) and partly of fixed factors (categorical demographic variables). The rationale of using regression analysis was that the distributions of the dependent variables (anxiety and depression) were fairly normal, where the Shapiro Wilk *p* values exceeded the 0.05 level of significance. Multicollinearity measures indicated that multicollinearity was not a cause for concern since the condition index was < 10 and the VIF was < 3. Moreover, the diagnostic tools indicated no serious problems with anomalous observations and model misspecifications.

A parsimonious model for anxiety was identified by using a backward elimination procedure. This model included a sole significant predictor (years of caring) which explained 20.4% of the total variance in the anxiety scores (Table 5). The regression coefficients (parameter estimates) indicated that carers who have been supporting a person with depression for 5 years or less were scoring, on average, 1.947 scale points more on anxiety than carers who have been caring for 11 and more years. Moreover, individuals who have been supporting the person with depression for 6–10 years were scoring, on average, 0.907 points more on anxiety than carers who have been providing care for over 11 years.

**Table 5:** Regressional analysis with anxiety as the dependent variable

Parameter	Regression coefficient, B	Standard error	*t* Value	*p* Value
Intercept	9.170	0.380	17.079	≤ .001
Years of caring category 1	1.947	0.483	4.968	≤ .001
Years of caring category 2	0.907	0.565	1.715	.09
Years of caring category 3	0^a^			

Dependant variable = anxiety, adjusted *R*^2^ = 20.4%, B = regression coefficient; 0^a^ = parameter set to 0; years of caring: category 1: ≤ 5 years; category 2: 6–10 years; category 3: 11+ years

A similar procedure was used to identify a parsimonious model for depression (Table 6). This model identified timeline chronicity, illness coherence and consequences (relatives) as significant predictors of depressions, explaining 56.8% of the total variance in the depression scores. As all the unstandardised regression coefficients have a positive value, there is a positive relationship between the predictors and the outcome variable. Thus, the increasing timeline chronicity beliefs, greater perceived impact on the carer and stronger beliefs in a lack of knowledge about depression are associated with higher depression scores. Consequently, as timeline chronicity increases by 1 unit, the depression score increases by 0.236 of a unit; as illness coherence increases by 1 unit, the depression score increases by 0.395 of a unit and for consequences (relative) with an increase of 1 unit depression scores increase by 0.256 of a unit. Similarly, no serious problems were encountered with multicollinearity, outliers and influential observations.

**Table 6:** Regressional analysis with depression as the dependent variable

Illness dimension subscales	Regression coefficient, B	Standard error of B	*t* Value	*p* Value
Intercept	− 8.844	1.951	− 4.534	≤ .001
Timeline chronicity	.236	.062	3.824	≤ .001
Illness coherence	.395	.117	3.362	.001
Consequences relative	.256	.087	2.924	≤ .01

Dependant variable = depression, adjusted *R*^2^ = 56.8%, B = regression coefficient

## Discussion

The present study is of theoretical importance as it represents the first application of the CSM to examine the illness perceptions of informal carers of persons with depression and identifies which of these perceptions are predictors of psychological well-being. Such information may serve as a guide when formulating interventions that target the psychological well-being of these carers. This information is also of importance as the illness perceptions and psychological well-being of informal carers have a strong influence on their supportive behaviour towards the care recipient [24].

### Identity

The symptoms which were strongly endorsed by the informal carers concur with a diagnosis of depression. However, whilst a loss of motivation, being withdrawn, worrying, loss of self-confidence, feeling worthless and loss of interest in personal care were strongly endorsed by informal carers as symptoms related to depression, a ‘lack of energy’, ‘gaining weight’ and ‘sleeping a lot’ were attributed by most of the carers to the medication taken.

### Causal attributes of depression

Causal attributes are of importance to the informal carers as they strive to understand the cause of the care recipient’s depression. Such causal beliefs are of importance as they influence the type of treatments that the individual perceives as necessary to control an illness [25]. The causal attributes most strongly endorsed by participants as a cause of depression were psychosocial aspects (e.g. stress and worry). The possible impact of chemicals as a cause of depression was endorsed by approximately half of the participants, which may influence the support provided to the care recipient to adhere to any medication prescribed. Furthermore, other causal triggers for depression such as traumatic events and the death of a loved one were poorly endorsed by participants. This finding highlights a misperception, whereby participants did not acknowledge traumatic events as causal triggers for depression. This may result in ‘problem minimisation’ during which informal carers play down the symptoms experienced by individuals following a traumatic event. This process may have a detrimental effect on the care recipient, who may be under the impression that s/he is not being taken seriously [24].

The identification of stress as a major causal trigger for depression maybe explained as it is perceived as a deleterious factor associated with a contemporary hectic lifestyle [25]. Stress as a causal trigger serves a dual purpose, having an external uncontrollable element and controllable internal elements [26]. Thus, the individual can avoid blaming him/herself or others, whilst at the same time seeking to control a recurrence [27]. In fact, only 3.2% of carers in the present study perceived their own behaviour as a causal trigger of the care recipient’s depression. Other causal items cited by respondents included personality, mental attitude and the patient’s own behaviour. The attribution of an illness to the care receiver’s personality has been demonstrated [27] to be associated with fewer positive appraisals in carers.

Participants in the present study also strongly AQendorsed a hereditary factor as a major causal trigger of depression. Blaming a hereditary factor as the cause for an illness can be associated with a family history of persons with the illness [28]. In addition, it has been demonstrated that by blaming a disease on heredity, persons tend to evade any blame for having or passing on their condition, as the disease is beyond their control [29].

### Illness perceptions

The illness perception profiles of study participants indicated that most perceived depression to be cyclical in nature and also perceived the treatment to be effective. In addition, depression was perceived as having consequences on the carer, but even more on the care recipient. According to Lobban et al. [12], perceptions of severe consequences in the informal carers of persons with a mental illness were associated with corresponding measures of distress and a sense of burden. These severe consequences have been documented in qualitative research studies which highlight the various challenges faced by carers of persons with depression relating to: financial difficulties and worries [30]; work constraints [31]; social isolation [6]; changes in family dynamics and relationships [6], and renouncing of leisure activities [30]. Moreover, findings in this study highlight that perceiving greater consequences in the relative was associated with perceptions of a chronic model of depression, poorer treatment control beliefs, a lower level of understanding about depression, the endorsing of more symptoms as associated with depression, a greater negative emotional impact for the carer and greater perceived consequences in the care recipient.

The present study also contributes to research to date by identifying that a poor understanding about depression (illness coherence), greater perceived consequences in the relative and a chronic model of depression were predictive of higher scores of depression in the informal carers. This finding lends support to the common-sense model [9] which posits that an individual’s perceptions are associated with outcomes, represented in this study by depression, as a measure of well-being. A poorer level of understanding may be predictive of higher depression scores as it is associated with perceptions of having less control over the care recipient’s mental health problems [12]. The impact of timeline chronicity beliefs on depression may be explained as longer timeline perceptions are associated with the carer perceiving poor personal control over the situation, with pessimism of ever returning back to a ‘normal life’ [25]. These findings should be interpreted within a context, where the carers of persons with depression are described as receiving little reprieve from their role [6]. In fact, findings in this study demonstrate that timeline chronicity beliefs are positively correlated with perceived consequences (patient and relative) and negatively associated with personal control beliefs in the informal carer.

The study also contributes to the academic literature by identifying ‘years of caring category’ as a predictor of anxiety with those carers providing 5 years or less of caring being identified as the most anxious. Thus, the initial phase following the diagnosis of a mental illness is stressful for the carers who often react with feelings of shock, confusion, fear and heightened vigilance [32]. Moreover, the encountering of unpredictable and unfamiliar situations in relation to the care recipient has been identified as increasing both stress and anxiety in these carers [5].

Despite all efforts to reduce possible flaws and rule out potential limitations in this research study, the researcher acknowledges the fact that certain methodological issues such as the cross-sectional design could have an influence on the conclusions made, as one cannot clarify the direction of relationships. For instance, it is impossible to determine whether depression leads the carers to interpret a disease as having negative consequences on their lives or whether experiencing negative consequences leads to depression. Future research using a longitudinal design could provide evidence-based statements regarding causal implications. In addition, the restriction of the sample to carers of persons who have received support in the community setting may have implications on the generalisability of the results to the general carer sample for persons with depression.

## Conclusion

This study highlights the importance of understanding the carers’ illness perceptions (as identified by the common-sense model) and their predictive values in relation to the psychological well-being of carers. Hence, interventions for such carers should target various illness perceptions highlighted in this study, such as the need for information, chronic timeline beliefs, misperceptions relating to causal attributes for depression and targeting the perceived consequences of providing support in informal carers. Psychoeducational interventions can be used to improve the carers’ understanding of the causes and treatment of depression and enhance the participants’ ability to cope with illness-related concerns relating to the mental illness [32–34]. Moreover, study findings have demonstrated an association between higher scores on understanding about depression with lower perceived consequences (for the patient and carer) and less emotional impact for the informal carers. Other approaches such as cognitive behaviour therapy are also of benefit as the one through the modification of the carers’ illness perceptions and appraisals [27], such as a restructuring of negative thoughts about the consequences of the disease.
